# Differential Modulation of the Phospholipidome of Proinflammatory Human Macrophages by the Flavonoids Quercetin, Naringin and Naringenin

**DOI:** 10.3390/molecules25153460

**Published:** 2020-07-29

**Authors:** Tiago A. Conde, Luís Mendes, Vítor M. Gaspar, João F. Mano, Tânia Melo, M. Rosário Domingues, Iola F. Duarte

**Affiliations:** 1 CICECO-Aveiro Institute of Materials, Department of Chemistry, University of Aveiro, 3810-193 Aveiro, Portugal; tiagoalexandreconde@ua.pt (T.A.C.); luisfilipemendes@ua.pt (L.M.); vm.gaspar@ua.pt (V.M.G.); jmano@ua.pt (J.F.M.); 2 LAQV-REQUIMTE, Mass Spectrometry Center, Department of Chemistry, University of Aveiro, 3810-193 Aveiro, Portugal; taniamelo@ua.pt; 3 CESAM, Department of Chemistry, University of Aveiro, 3810-193 Aveiro, Portugal

**Keywords:** macrophages, phospholipids, flavonoids, immunomodulation, inflammation, lipidomics, mass spectrometry

## Abstract

The immunomodulatory activity of flavonoids is increasingly appreciated. Macrophage phospholipids (PLs) play crucial roles in cell-mediated inflammatory responses. However, little is known on how these PLs are affected upon flavonoid treatment. In this work, we have used mass-spectrometry-based lipidomics to characterize the changes in the phospholipidome of proinflammatory human-macrophage-like cells (THP-1-derived and LPS+IFN-γ-stimulated) incubated with non-cytotoxic concentrations of three flavonoids: quercetin, naringin and naringenin. One hundred forty-seven PL species belonging to various classes were identified, and their relative abundances were determined. Each flavonoid displayed its own unique signature of induced effects. Quercetin produced the strongest impact, acting both on constitutive PLs (phosphatidylcholines, phosphatidylethanolamines and sphingomyelins) and on minor signaling lipids, such as phosphatidylinositol (PI) and phosphatidylserine (PS) species. Conversely, naringin hardly affected structural PLs, producing changes in signaling molecules that were opposite to those seen in quercetin-treated macrophages. In turn, albeit sharing some effects with quercetin, naringenin did not change PI and PS levels and interfered with a set of phosphatidylcholines distinct from those modulated by quercetin. These results demonstrate that flavonoids bioactivity involves profound and specific remodeling of macrophage phospholipidome, paving the way to future studies on the role of cellular phospholipids in flavonoid-mediated immunomodulatory effects.

## 1. Introduction

Inflammation is a complex physiological response to potentially harmful stimuli, with a fundamental role in protecting and restoring homeostasis [1]. However, non-resolving inflammatory responses can have deleterious consequences. Low-grade chronic inflammation has been implicated in the pathogenesis of several diseases (e.g., atherosclerosis, type 2 diabetes mellitus, rheumatoid arthritis) [2], as well as in the rejection of transplant organs and medical implants [3]. Macrophages are phagocytic innate immune cells with a crucial role in inflammation [1,4]. Depending on the microenvironmental signals, they polarize into different activation states, from proinflammatory (M1-like) to anti-inflammatory (M2-like), whose balance is crucial for an adequate onset and a timely resolution of inflammation [5]. Hence, modulation of macrophage polarization has emerged as an attractive strategy in various medical contexts [6,7].

Several plant phenols display strong anti-inflammatory activity in vitro and in vivo [8], which makes them good candidates for tackling chronic inflammation and its associated diseases. Flavonoids are natural polyphenols widely present in plant-derived foods and beverages, whose basic structure (abbreviated as C6-C3-C6) consists of a 15-carbon skeleton composed of two phenyl rings and an oxygen-containing pyran ring [9]. While most flavonoids were initially valued for their antioxidant activity, their ability to modulate immune cells’ inflammatory responses has been increasingly recognized [10]. Flavonoids are known to exert their anti-inflammatory action via multiple mechanisms, which include (1) promoting M1- to M2-like transition [11], (2) reducing the production of proinflammatory cytokines, mainly through inhibition of nuclear factor kappa-light-chain-enhancer of activated B cells (NF-kB) and mitogen-activated protein Kinase (MAPK) signaling pathways and (3) inhibiting cellular enzymes involved in arachidonic acid release and conversion to prostaglandins and leukotrienes [10]. Recently, the modulation of immune cells’ intracellular metabolism has also been associated with the anti-inflammatory activity of flavonoids. These compounds were proposed to suppress the glycolysis-promoting PI3K/Akt/mTOR pathway in T helper (Th) inflammatory cells, leading to induction of T regulatory cells [12]. Moreover, flavonoid-mediated metabolic reprogramming of macrophages has been previously reported by us [13]. In that work, we found that quercetin, naringenin and, to a lesser extent, naringin significantly modulated the polar metabolome of prepolarized M1 macrophages, mainly towards downregulation of glycolysis and reprogramming of the tricarboxylic acid (TCA) cycle. Moreover, changes in choline metabolites suggested membrane modification to be involved in cellular responses to flavonoids. Following on those results, in the present study, we investigate the impact of quercetin, naringin and naringenin on the phospholipidome of proinflammatory macrophages.

Phospholipids are not only the main building blocks of cell membranes, but they are also critically involved in cell signaling and membrane remodeling processes required for cells to perform multiple functions. Macrophage phospholipids play crucial roles in cell-mediated inflammatory responses, namely by providing precursors for the synthesis of potent lipid mediators, including proinflammatory eicosanoids, such as prostaglandins, and specialized pro-resolving mediators (SPMs) like resolvins [14]. Moreover, some phospholipids (mainly plasmalogens) act as physiological antioxidants [15,16], while others like phosphoinositides are important signaling molecules involved in the regulation of cytokine production [17]. Previous studies have documented significant changes in the phospholipid composition of macrophages incubated with M1-polarizing stimuli (bacterial lipopolysaccharide and/or interferon-γ) [18,19,20,21]. While those reports highlighted a close link between macrophage lipid profiles and inflammation, little is known on the ability of flavonoids to modulate macrophage lipid composition and metabolism. To address this question, in the present study, we performed a large-scale characterization of variations in the phospholipid profile of proinflammatory macrophages treated with quercetin, naringin and naringenin, using a mass-spectrometry-based lipidomics approach. Such untargeted, holistic screening of the cells’ phospholipidome has great potential to aid in interpreting flavonoid-mediated anti-inflammatory action via the biosynthesis and modification of key lipid mediators.

## 2. Results

### 2.1. Phospholipidome of Proinflammatory Macrophages

**Figure 1:**
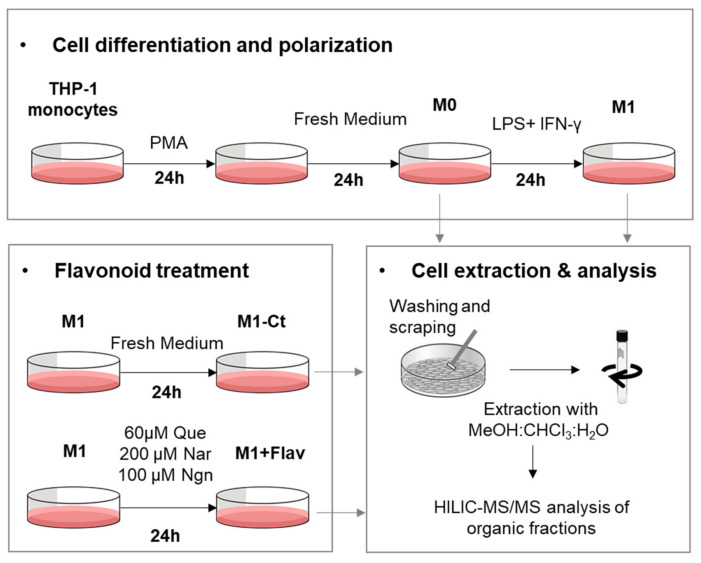
Schematic representation of cell culture and sample collection for HILIC-MS/MS analysis (Flav, flavonoid; Que, quercetin; Nar, naringin; Ngn, naringenin).

The phospholipid (PL) composition of THP-1-derived macrophage-like cells, activated by proinflammatory stimuli (LPS+IFN-γ) and subsequently treated with different flavonoids, was profiled using HILIC-LC-MS/MS, according to the experimental setup schematically represented in Figure 1. In total, we identified 147 PL molecular ions, which may arise from 199 possible different PL species, as detailed in Supplementary Table S1. The identified species belong to eight PL classes: phosphatidylcholines (PCs; comprising diacyl and alkyl/alkenyl-acyl species), lysophosphatidylcholines (LPCs), phosphatidylethanolamines (PEs; comprising diacyl and alkyl/alkenyl-acyl species), lysophosphatidylethanolamines (LPEs), phosphatidylglycerols (PGs), phosphatidylinositols (PIs), phosphatidylserines (PSs) and sphingomyelins (SMs). 

**Figure 2:**
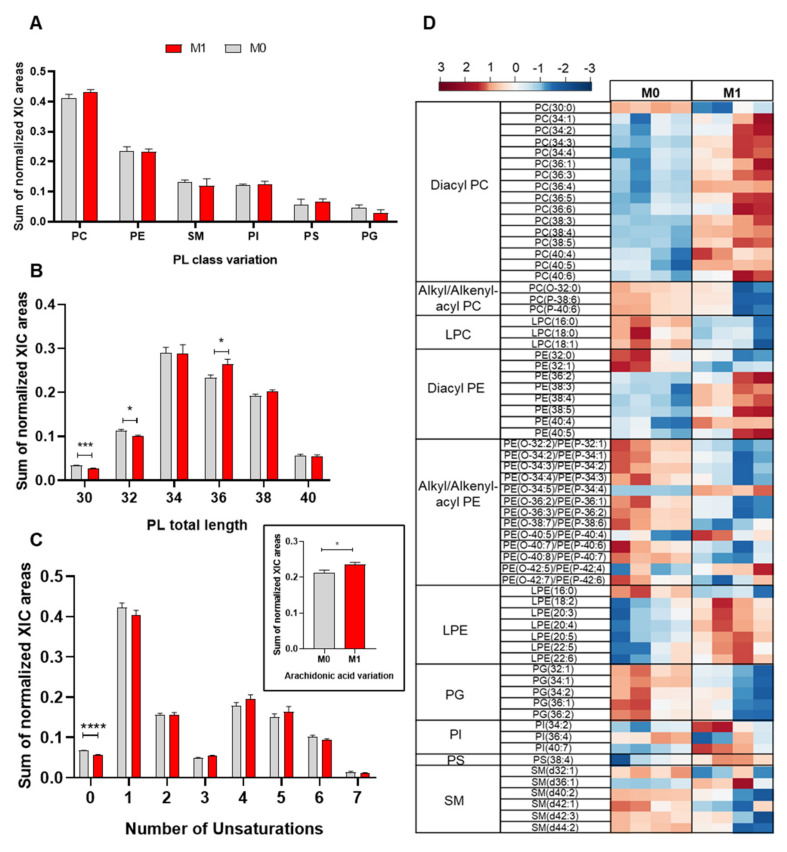
Phospholipidome of THP-1-derived macrophage-like cells, unstimulated (M0) and activated with LPS+IFN-γ (M1): (**A**) Relative abundances of main phospholipid (PL) classes (PC, phosphatidylcholine; PE, phosphatidylethanolamine; SM, sphingomyelin; PI, phosphatidylinositols; PS, phosphatidylserine; PG, phosphatidylglycerol). The PC class comprises diacyl-, alkyl/alkenyl-acyl and lysophosphatidylcholines, and the PE class comprises diacyl-, alkyl/alkenyl-acyl and lysophosphatidylethanolamines. (**B**) Number of carbons in PL fatty acyl chains. (**C**) Number of double bonds in PL fatty acyl chains. The inset shows the amount of PLs containing arachidonic acid (AA, 20:4). * *p* < 0.05, *** *p* < 0.001, **** *p* < 0.0001. (**D**) Heatmap representing the relative abundance of PL species found to be most important (VIP > 1) for discriminating between M0 and M1 samples through PLS-DA. The peak intensity values were normalized by total area, log-transformed and autoscaled.

As a first approach to assess the impact of proinflammatory activation on the cells’ phospholipidome, we compared the relative abundances of the different PL classes in M0 and M1 macrophages (LPS+IFN-γ-stimulated) by considering the summed integrals of all peaks belonging to the same class. As shown in Figure 2A, proinflammatory activation appeared to increase the relative abundance of PC, while decreasing PG, although these differences did not reach statistical significance. Moreover, proinflammatory macrophages displayed significantly higher relative abundance of PL species with 36 carbons in their FA chains, together with lower levels of PLs with shorter FA chains (30 and 32 carbons) (Figure 2B). Regarding the unsaturation degree, upon proinflammatory activation, PL species containing saturated and monounsaturated fatty acids (MUFA) decreased, while those with polyunsaturated fatty acids (PUFA) increased (Figure 2C). Accordingly, PLs containing arachidonic acid (20:4) were increased in M1 vs. M0 macrophages (inset in Figure 2C).

To identify the individual PL species affected by LPS+IFN-γ stimulation, multivariate analysis was applied. The two sample groups (M0 and M1) were clearly separated in the PCA and PLS-DA scores scatter plots (Supplementary Information Figure S1). The variables with higher importance in the PLS-DA model (VIP > 1) were then represented in a heatmap (Figure 2D). Despite some intragroup variability, several PLs showed very reproducible and markedly different relative abundances in M0 and M1 macrophages. Within PC species, which represented over 30% of the discriminating PLs (VIP > 1), 15 diacyl PCs were increased; meanwhile, one diacyl (30:0) and three alkyl/alkenyl-acyl (ether) PCs, together with three LPCs (all with saturated or monounsaturated FA), were decreased in M1 vs. M0 macrophages. As for variations in PE species, which represented over 40% of discriminatory features, they comprised increases in six diacyl PEs, four ether PEs and six LPEs and decreases in two diacyl PEs, nine ether PEs and one LPE. Notably, most PE species displaying higher relative abundance in proinflammatory macrophages contained longer, polyunsaturated FAs, whereas those with decreased levels contained shorter FA chains with lower unsaturation. Additional PLs with VIP > 1 comprised five PGs (all decreased in M1 vs. M0), three PIs (two increased), one PS (increased) and five SMs (four decreased). 

### 2.2. Flavonoid-Induced Phospholipidome Remodeling

**Figure 3:**
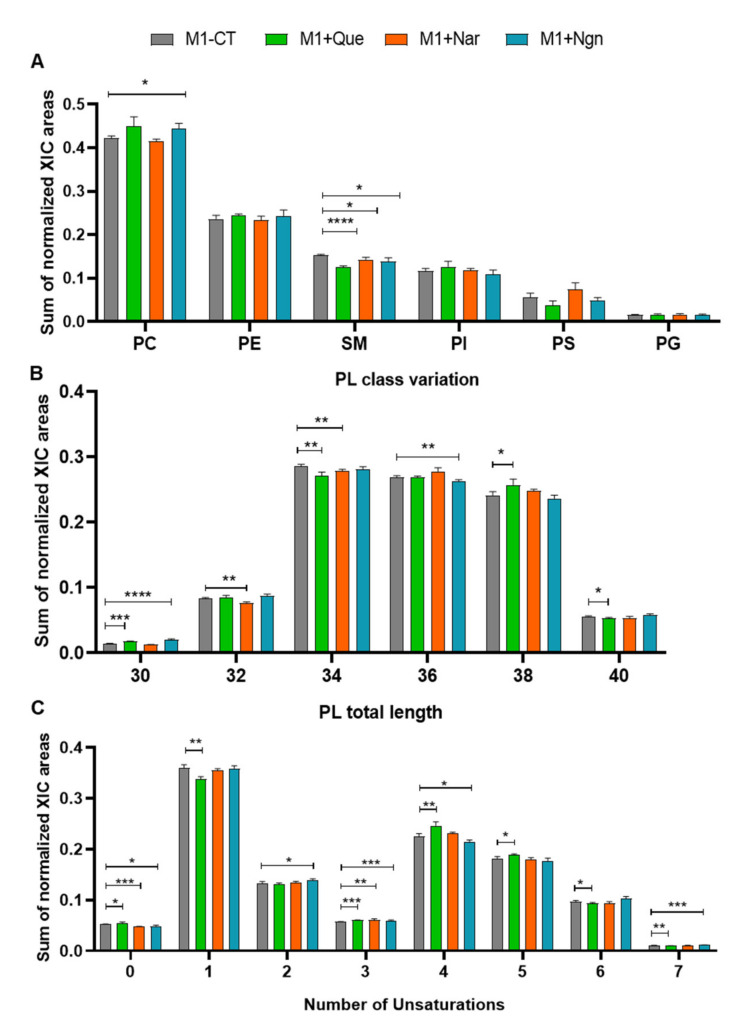
Phospholipidome of proinflammatory THP-1-derived macrophage-like cells, incubated for 24 h in flavonoid-free medium (M1-Ct) or treated with quercetin (M1+Que), naringin (M1+Nar) or naringenin (M1+Ngn): (**A**) Relative abundances of main phospholipid (PL) classes (PC, phosphatidylcholine; PE, phosphatidylethanolamine; SM, sphingomyelin; PI, phosphatidylinositol; PS, phosphatidylserine; PG, phosphatidylglycerol). The PC class comprises diacyl-, alkyl/alkenyl-acyl and lysophosphatidylcholines, and the PE class comprises diacyl, alkyl/alkenyl-acyl- and lysophosphatidylethanolamines. (**B**) Number of carbons in PL fatty acyl chains. (**C**) Number of double bonds in PL fatty acyl chains. * *p* <0.05, ** *p* < 0.01, *** *p* < 0.001, **** *p* < 0.0001.

Proinflammatory (M1) prepolarized macrophage-like cells were incubated for 24 h with non-cytotoxic concentrations of quercetin, naringin or naringenin. As reported in our preceding study [13], all flavonoids induced lower production of proinflammatory cytokines, mainly TNF-α, by prepolarized M1 macrophages, while naringenin further increased the production of the anti-inflammatory chemokine CCL17. In the present study, we focus on the phospholipidome changes accompanying flavonoid treatment. In terms of overall variations in PL classes (Figure 3A), the most prominent changes were seen for PCs (increased by quercetin and naringenin), SMs (decreased by all flavonoids, especially by quercetin) and PSs (decreased by quercetin, increased by naringin). Fatty acyl chain length and unsaturation degree were also modulated by the three flavonoids (Figure 3B,C), while the content in arachidonic acid did not change significantly. 

**Figure 4:**
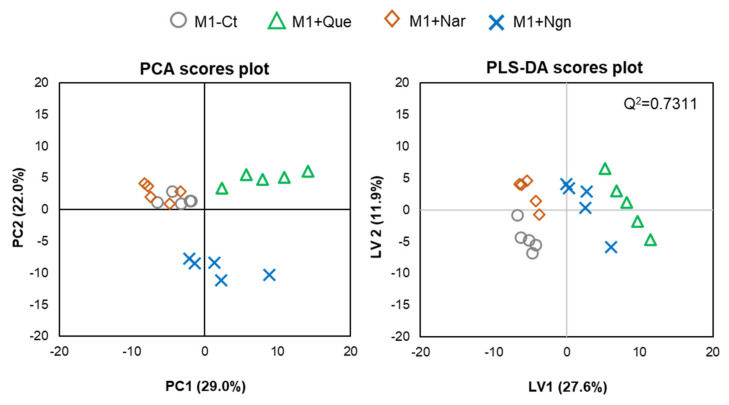
Multivariate analysis of LC-MS data collected for M1 macrophages exposed to different flavonoids: PCA (left) and PLS-DA (right) scores scatter plots.

**Figure 5:**
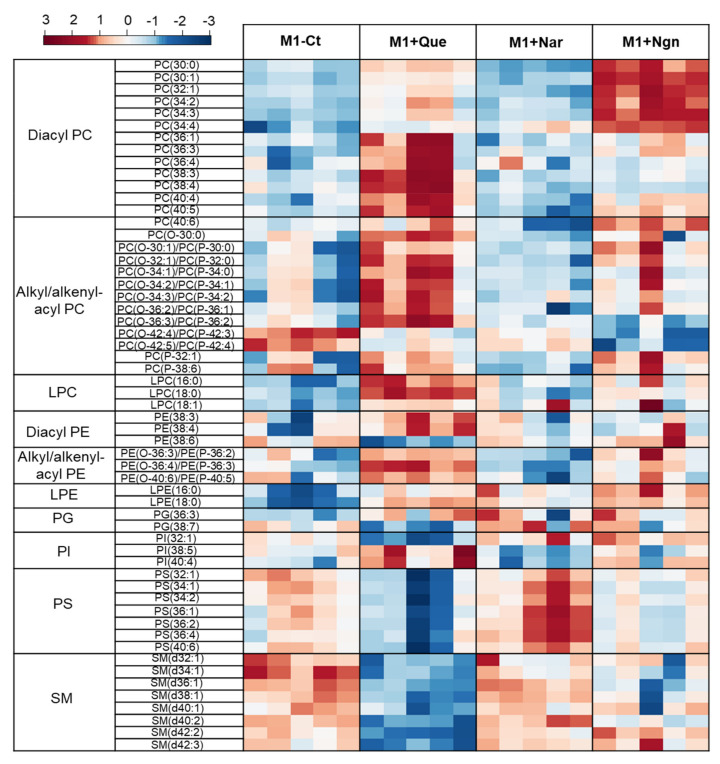
Heatmap representing the relative abundance of PL species found to be most important (VIP > 1) for discriminating between control M1 proinflammatory macrophages (M1-Ct) and flavonoid-treated M1 cells. The peak intensity values were normalized by total area, log-transformed and autoscaled.

Multivariate analysis of all the PL species identified further helped to reveal the specific effects produced by each flavonoid. In the PCA scores scatter plot obtained for the four groups compared (Figure 4, left), quercetin- and naringenin-treated samples are clearly separated from one another and from M1-Ct, whereas naringin-treated cells are less separated from controls. This is corroborated by PLS-DA, where the four groups are discriminated with high robustness (Q2 0.93 for the first two latent variables (LVs)), distributing along LV1 in the scores scatter plot (Figure 4, right). The most important variations contributing to class discrimination (VIP > 1) were then represented in a summary heatmap (Figure 5). Quercetin and naringenin induced pronounced changes in the relative abundance of several diacyl PCs, while naringin had little effect. Compared to M1-Ct, quercetin-treated cells displayed higher levels of PCs containing longer FA chains (36–40 carbons), whereas naringenin caused an increase in shorter FA-containing PLs (30–34 carbons). As for alkyl/alkenyl-acyl PCs, quercetin induced pronounced increases in eight species, while naringenin caused two others to decrease. The relative abundances of 3 LPC species were also consistently upregulated by quercetin. Regarding PE species, quercetin and, to a lesser extent, naringenin caused increases in the relative levels of two diacyl PEs and three ether PEs, while two LPEs were more strongly increased by naringenin. A few PG and PI species were also highlighted as having VIP > 1 for at least one of the flavonoids tested. For instance, quercetin downregulated PI (32:1) and upregulated PI (38:5) and PI (40:4), while the opposite effect was produced by naringin. These two flavonoids also affected the levels of several PS species differently, with quercetin causing consistent decreases and naringin raising their levels. Naringenin hardly affected PS. Finally, eight SMs strongly responded to quercetin by decreasing their relative abundance in comparison to M1-Ct cells.

## 3. Discussion

A deeper understanding of the immunomodulatory activity of widely available flavonoids may contribute to expand their range of applications as nutraceuticals or anti-inflammatory drugs. Lipids are important players in immune-cell-mediated inflammatory responses, and hence, in this work, we have looked at how three different flavonoids modulate the phospholipidome of proinflammatory human macrophages. An initial comparison of LPS+IFN-γ-stimulated cells (M1 macrophages) with unstimulated (M0) macrophages corroborated the previously reported shift in the PL composition of THP-1-derived macrophages from short-chain saturated/monounsaturated FAs to long-chain polyunsaturated FAs upon M1 polarization [19]. In line with that report, we also found an increase in PL species containing arachidonic acid (20:4), which likely reflects AA redistribution in PLs to support the biosynthesis of bioactive eicosanoids mediating the inflammatory response. We have then focused on flavonoid-induced phospholipidome remodeling of M1 prepolarized macrophage-like cells. Amongst the tested compounds, quercetin and naringenin triggered more pronounced alterations than naringin, in line with our previous report on the polar metabolome [13]. Notably, the lipidomic signatures of macrophages incubated with the three flavonoids differed not only in magnitude but also in a qualitative manner, providing evidence for specific, finely tuned cellular responses to structurally similar compounds.

The relative abundances of several PC species were strongly and differentially affected by quercetin and naringenin, while showing milder changes upon naringin treatment. Quercetin increased the levels of eight diacyl PCs containing longer and more polyunsaturated FAs, while naringenin upregulated diacyl PCs with shorter and less unsaturated FAs. Additionally, while several of these changes were common to proinflammatory activation, naringenin caused diacyl PC (30:0) to increase, contrarily to the variation induced in M1 vs. M0 macrophages. Phosphatidylcholines are major components of mammalian cell membranes, with a key role in regulating membrane properties like the position of proteins, membrane fluidity and permeability [22]. Moreover, recent findings indicate that the role of PCs in mammalian cells extends well beyond structural functions, encompassing regulation of specific genes and signaling pathways [23]. For instance, 1-palmitoyl-2-oleoyl-sn-glycerol-3-phosphocholine (16:0/18:1 PC, POPC) acts as a ligand for the peroxisome proliferator-activated receptor PPARα, which interacts with the transcription factor NF-κB, inhibiting its action [24]. Oxidized phospholipids, such as 1-palmitoyl-2-arachidonyl-snglycero-3-phosphorylcholine (OxPAPC), have also been shown to inhibit TLR signaling in macrophages via competitive binding to accessory proteins that interact with bacterial lipids, including CD14, LBP and MD2 [25,26]. Notably, studies have shown that cellular receptors are able to distinguish PCs with different FA residues [23]. Hence, the fact that the three flavonoids, especially quercetin and naringenin, differentially modulated the levels of specific PCs supports the idea that these species may have multiple, unpredictable roles, stressing the importance of carrying out comprehensive lipidomics studies.

The levels of several alkyl-acyl and alkenyl-acyl (plasmalogen) PCs were also markedly affected by flavonoids. Consistent increases were observed in eight of these species upon treatment with quercetin, while naringenin induced decreased levels of two other alkyl/alkenyl-acyl PCs. Additionally, three ether PEs were upregulated by the two flavonoids, especially by quercetin. Naringin modulated PC and PE ether lipids to a lesser extent and towards lower relative abundances, compared to M1-Ct. The functions of ether PLs in mammalian cells are still largely unknown, although there is increasing awareness of their importance in the regulation of membrane homeostasis and in cell signaling [27]. For instance, in a mouse model of atopic dermatitis, ceramide levels were decreased in the skin barrier, while supplemental feeding with ether lipids helped to alleviate skin inflammation [28]. Moreover, plasmalogens may function as endogenous antioxidants by protecting unsaturated membrane lipids from oxidation and scavenging reactive oxygen species (ROS) [29]. Hence, a possible link between their modulation and the antioxidant properties of flavonoids should not be ruled out. 

The lysoglycerophospholipids LPC 16:0, 18:0 and 18:1 and LPE 16:0, which showed depleted levels in M1 vs. M0 macrophages, increased their relative abundance in flavonoid-treated proinflammatory cells. Previous studies have proposed LPCs to be inflammatory mediators acting via multiple mechanisms, including the perpetuation of macrophage proinflammatory phenotype [30]. On the other hand, tumor cells were reported to quickly degrade saturated and monounsaturated LPCs, subsequently incorporating the released FAs into their membranes, which increases membrane rigidity and reduces the cells’ migratory potential [31]. Given the established similarity between the metabolism of tumor cells and proinflammatory macrophages [32], it seems reasonable to speculate that M1 macrophages also exhibit an increased metabolism of lysoglycerophospholipids, thereby displaying decreased levels compared to M0 controls, whereas quercetin and naringenin oppose this effect.

Flavonoids additionally modulated PI and PS species, which are widely recognized as signaling molecules [33,34,35]. In particular, three PIs (32:1, 38:5 and 40:4) were differentially modulated by quercetin and naringin (while naringenin had no relevant effect). Among other roles, PIs act as substrates of phosphatidylinositol 3-kinases (PI3K), which catalyze their phosphorylation to phosphatidylinositol phosphates (PIP), known to act as second messengers in multiple cellular processes [36]. The ability of flavonoids like quercetin and fisetin to inhibit the PI3K signaling pathway has been linked mostly to their anticancer activity, while its relation to immune responses is still unclear [12]. Our results further showed that several PS species decreased upon quercetin treatment, while displaying increased levels in naringin-treated cells. Hence, like PIs, this class of signaling PL, previously shown to have anti-inflammatory effects both in vitro and in vivo [37], was also differentially affected by the three flavonoids studied. Finally, quercetin, but not the other flavonoids, significantly decreased the levels of several sphingomyelins, following a similar trend to that seen for M1 vs. M0 macrophages, with the exception of SM 36:1 (increased upon proinflammatory activation and decreased in quercetin-treated M1 cells). These sphingolipids are abundant structural components of membranes, while also acting as precursors of proinflammatory signaling molecules, such as ceramide, ceramide-1-phosphate and sphingosine-1-phosphate [38]. SM cellular content can also directly affect inflammatory signaling, with reduced SM content, caused by pharmacologic inhibition of sphingomyelin synthase, having been reported to render macrophages less responsive to LPS [39]. Hence, our findings of decreased SM levels both upon proinflammatory activation and in quercetin-treated cells are in line with the notion that SM-derived ceramides may potentiate or attenuate inflammatory signaling, depending on the stimulus considered [40].

While this study was designed to assess flavonoid-induced modulation of proinflammatory macrophages, the impact of the studied compounds towards unstimulated (M0) and anti-inflammatory, proresolution (M2-like) macrophages should be addressed in future work. Indeed, based on the close link between these cells’ phenotype and metabolism [41], we expect flavonoid-induced metabolic changes (including phospholipidome changes) to be highly dependent on the initial cellular activation state. Hence, a comprehensive view of flavonoids’ effects towards differentially activated macrophages could potentially reveal metabolic alterations implied not only in the reversal of proinflammatory activity, but also in the promotion of a proresolution phenotype. Furthermore, transcription and/or expression of genes related to phospholipid metabolism should provide relevant insights to help explain the alterations observed herein. Finally, it should be acknowledged that the flavonoid concentrations used in this work (60–200 µM) were 1 or 2 orders of magnitude higher than the maximal flavonoid concentrations found in human plasma [42,43]. While we have used the highest concentration of each flavonoid not affecting cell viability in order to trigger the potentially strongest responses, it will be important to assess, in future studies, how flavonoid concentrations closer to physiological levels modulate macrophage phenotype and metabolism.

## 4. Materials and Methods 

### 4.1. Test Compounds and Reagents

Stock solutions of quercetin (97% purity, Alfa Aesar, Ward Hill, MA, USA), naringenin (≥95% purity, Merck-Sigma Aldrich, St. Louis, MO, USA) and naringin (≥95% purity, Merck-Sigma Aldrich, St. Louis, MO, USA) were prepared with 99.5% DMSO at a concentration of 80 mM. Phorbol 12-myristate 13-acetate (PMA, Merck-Sigma Aldrich, St. Louis, MO, USA) was prepared in 99.5% DMSO at a concentration of 100 μg/mL. Powdered LPS (Merck-Sigma Aldrich, St. Louis, MO, USA) was dissolved in Gibco Water for Injection (WFI) at a concentration of 1 mg/mL. Interferon-γ (IFN-γ, Biolegend, San Diego, CA, USA) was kept in the original formulation at a concentration of 0.1 mg/mL. All solutions were kept at −20 °C and protected from light.

### 4.2. Differentiation and Polarization of THP-1 Cells

Human monocytic THP-1 cells (ATCC, Virginia, USA) were cultured in Roswell Park Memorial Institute (RPMI 1640) culture medium that contained 10% heat-inactivated fetal bovine serum (FBS, South America Origin, Alfagene, Carcavelos, Portugal) and 1% penicillin/streptomycin and was supplemented with 2.0 g/L sodium bicarbonate. Cells were maintained in culture in a humidified atmosphere at 37 °C and 5% CO_2_. THP-1 monocytes were differentiated into macrophages (M0) by 24-h incubation with 50 ng/mL PMA in RPMI medium, which was followed by a 24-h rest in new standard complete medium. Proinflammatory macrophage activation was carried out by incubation with 20 ng/mL IFN-γ and 100 ng/mL LPS for 24 h, and it was confirmed by increased production of proinflammatory cytokines, as reported in our previous work [13].

### 4.3. Incubation of Proinflammatory THP-1-Derived Macrophages with Flavonoids

THP-1 monocytes (passages 15–17) were seeded at a density of 1 × 10^6^ cells/mL in cell-culture-treated 100-mm Petri dishes, differentiated and polarized to proinflammatory M1 cells as described above. Then, macrophages were treated with quercetin (60 µM), naringin (200 µM), naringenin (100 µM), or medium alone (M1-Ct) for a period of 24 h. The flavonoid concentrations used were the highest concentrations tested that did not cause significant reduction of cell viability, as determined by the Alamar Blue assay (results reported in Mendes et al. [13]). 

### 4.4. Cell Extraction for Lipidomics

Lipids were extracted according to a modified Bligh and Dyer extraction protocol [13,44], using methanol:chloroform:water (1:1:0.7) to obtain aqueous and organic phases. Firstly, the medium was removed, and the cells were washed four times with PBS. Then, 1 mL methanol 80% (*v*/*v*) (HPLC grade, Sigma Aldrich) was added to the dish to quench metabolic activity, and cells were scrapped off. The cell suspension was transferred into a glass tube containing 0.5-mm glass beads (to aid cell breakage). After 2 min of vortexing, 400 µL of cold chloroform (HPLC grade, VWR) were added to each tube, followed by vortexing and another addition of 400 µL of chloroform and 360 µL cold milli-Q water. Samples were vortexed again and left to rest on ice for 20 min. Then, after centrifugation at 3000 × *g* for 10 min, the bottom organic phase was transferred into an amber glass vial, and the remaining sample was re-extracted with 400 µL of chloroform to collect the organic phase again and add it to the previous vial. Organic extracts containing lipids, obtained from five cell-sample replicates for each condition, were then dried under a nitrogen gas stream and stored at −80 °C.

### 4.5. Phospholipid Quantification by Phosphorous Measurement

In order to determine the total phospholipid (PL) content in each lipid extract, the phosphorous assay was performed according to Bartlett and Lewis [45]. Phosphate standards from 0.1 to 2 μg of phosphorous (P) were prepared from a monosodium phosphate solution (100 µg/mL, NaH_2_PO_4_.2H_2_O). Dried aliquots of samples and standards were resuspended in 125 µL of perchloric acid. Samples were then heated at 180 °C in a heating block (Stuart, UK) for 40–60 min, followed by cooling at room temperature. Thereafter, 825 μL of milli-Q water, 125 μL of 2.5% ammonium molybdate solution and 125 μL of 10% ascorbic acid were added to samples and standards, which were vortexed after each addition and incubated for 10 min at 100 °C in a water bath. After cooling in a cold-water bath, the absorbance of samples and standards was measured at 797 nm in a microplate reader (Multiscan 90, ThermoScientific). Four independent measurements were performed for each sample.

### 4.6. Phospholipid Analysis by Hydrophilic Interaction Liquid Chromatography Coupled to High-Resolution Tandem Mass Spectrometry (HILIC-MS/MS)

Dried samples were dissolved in dichloromethane to obtain a PL concentration of 1 µg/µL. A volume of 5 µL was taken from each sample and transferred to an appropriate vial, followed by addition of 91 µL of a solvent system consisting of two mobile phases in a proportion of 60% eluent B (60% acetonitrile, 40% methanol and 1 mM of ammonium acetate) and 40% eluent A (50% acetonitrile, 25% methanol, 25% water and 1 mM of ammonium acetate) and 4 µL of an internal standard mixture containing PC (14:0/14:0), 0.02 µg; PE (14:0/14:0), 0.02 µg; PG (14:0/14:0), 0.012 µg; PI (16:0/16:0), 0.08 µg; PS (14:0/14:0), 0.04 µg; PA (14:0/14:0), 0.08 µg; LPC (19:0), 0.02 µg; Ceramide (d18:1/17:0), 0.04 µg; SM (d18:1/17:0), 0.02 µg; and CL (14:0/14:0/14:0/14:0), 0.08 µg. 

Phospholipids were separated through hydrophilic interaction liquid chromatography (HILIC-LC) using an Ascentis Si HPLC Pore (15 cm × 1.0 mm, 3 µm; Sigma-Aldrich) and a high-performance liquid chromatography (HPLC) system (Ultimate 3000 Dionex, Thermo Fisher Scientific, Bremen, Germany) with an autosampler coupled online to the Q-Exactive hybrid quadrupole Orbitrap mass spectrometer (Thermo Fisher Scientific, Bremen, Germany). An aliquot of 5 µL of each sample mixture was injected into the HPLC column at a flow rate of 40 µL/min and a temperature of 30 °C. Initially, 40% of mobile phase A was held isocratically for 8 min, followed by a linear increase to 60% of mobile phase A for 7 min, a 5 min maintenance period and the return to initial conditions within 5 min. A 10-min interval was given between injections to allow return to equilibrium. The mass spectrometer with a Q-Exactive orbitrap and a heated electrospray ionization (HESI) operated simultaneously in positive mode (voltage of 3 kV) and negative mode (voltage of −2.7 kV). The sheath gas flow rate was maintained at 15 units and the capillary temperature was 250 °C, while S-lenses RF was of 50 units and the probe’s temperature was 100 °C. Mass spectral acquisition method was at full scan in a scale of *m*/*z* values of 200–1600, with a resolution of 70.000, automatic gain control of 1 × 10^6^ and 2 microscans. The 10 most abundant ions were selected for ion fragmentation at collision cell HCD. Collision energy varied between 25, 30 and 35 eV. The tandem mass spectra (MS/MS spectra) were obtained at a resolution of 17.500, automatic gain control of 1 × 10^5^, 1 microscan and an isolating window of 1 *m*/*z*. Ion selection was limited to 2 × 10^4^ counts. Maximum accumulated ions were established at 100 ms for MS spectra and 50 ms for MS/MS spectra. Dynamic exclusion was defined as 60 s.

Data acquisition was performed using the software Xcalibur v3.3 (Thermo Fisher Scientific, USA). Identification of the different lipid species was performed based on retention time, exact mass measurements of the ions assigned as lipid species (less than 5 ppm) and on manual interpretation of the MS/MS spectra. All the MS/MS fragmentation patterns characteristic of the lipid classes analyzed in the present study were acquired in positive and negative ion modes, as reported previously [46,47], and representative examples can be found in Supplementary Figures S1–S7. After identification, quantification of molecular species was performed through integration of chromatographic peaks. LC-MS data were processed and integrated using the software MZmine v2.32. The software allows for filtering and smoothing, peak detection, peak processing and assignment against an in-house database [48]. During the processing of raw data acquired in full MS mode, all the peaks with raw intensity lower than 1 × 10^5^ were excluded. Relative quantification was performed by exporting peak area values into a computer spreadsheet (Excel, Microsoft, Redmond, WA, USA). For normalizing the data, peak areas of the extracted-ion chromatograms (XICs) of each lipid molecular ion were divided by the sum of total XIC areas of the identified lipid molecular ions.

### 4.7. Statistical Analysis

Multivariate analysis of data matrices (normalized peak areas of lipid molecular ions in the different samples) was performed using Metaboanalyst [49]. The data was log-transformed and autoscaled (i.e., scaling to unit variance (UV)) before principal component analysis (PCA) and partial least squares discriminant analysis (PLS-DA). The results were visualized in scores scatter plots and variable importance (VIP) plots showing the top variables contributing to sample discrimination. The normalized, log-transformed and UV-scaled areas of species with VIP larger than 1 were then represented in heatmaps to enable their relative abundance to be compared between sample groups. Heatmaps were generated using the R software version 3.4.1 (R Foundation for Statistical Computing, Vienna, Austria). 

## 5. Conclusions

This work demonstrates that the flavonoids quercetin, naringin and naringenin differentially modulate the phospholipidome of proinflammatory THP-1-derived macrophages. Quercetin produced the strongest impact, acting both on more abundant constitutive PLs (PC, PE and SM classes) and minor signaling lipids, like a few PI and PS species. On the other hand, naringin hardly affected structural PLs, producing changes in PI and PS species that were opposite to those seen in quercetin-treated macrophages. In turn, albeit sharing some effects with quercetin, naringenin did not change PI and PS levels and interfered with a set of PCs distinct from those modulated by quercetin. These results highlight macrophage plasticity and stimuli-dependent responses, contributing to revealing the intricate bioactivity of flavonoids and exploring their pharmacological potential in modulating proinflammatory processes.

## Supplementary Materials

The following are available online. Figure S1: Identification of phosphatidylethanolamine (PE); Figure S2: Identification of phosphatidylcholine (PC); Figure S3: Identification of phosphatidylinositol (PI); Figure S4: Identification of phosphatidylglycerol (PG); Figure S5: Identification of phosphatidylserine (PS); Figure S6: Identification of sphingomyelin (SM); Figure S7: Identification of lyso-phosphatidylcholine (LPC); Figure S8. Multivariate analysis of LC-MS data collected for M0 (grey circles) and M1 (red squares) macrophages.

## References

[1] Ferrero-Miliani L., Nielsen O.H., Andersen P.S., Girardin S.E. (2007). Chronic inflammation: Importance of NOD2 and NALP3 in interleukin-1β generation. Clin. Exp. Immunol..

[2] Prasad S., Aggarwal B.B. (2014). Chronic Diseases Caused by Chronic Inflammation Require Chronic Treatment: Anti-inflammatory Role of Dietary Spices. J. Clin. Immunol..

[3] Brown B.N., Londono R., Tottey S., Zhang L., Kukla K.A., Wolf M.T., Daly K.A., Reing J.E., Badylak S.F. (2012). Macrophage phenotype as a predictor of constructive remodeling following the implantation of biologically derived surgical mesh materials. Acta Biomater..

[4] Spiller K.L., Wrona E.A., Romero-Torres S., Pallotta I., Graney P.L., Witherel C.E., Panicker L.M., Feldman R.A., Urbanska A.M., Santambrogio L. (2016). Differential gene expression in human, murine, and cell line-derived macrophages upon polarization. Exp. Cell Res..

[5] Atri C., Guerfali F.Z., Laouini D. (2018). Role of human macrophage polarization in inflammation during infectious diseases. Int. J. Mol. Sci..

[6] Liu Y.-C., Zou X.-B., Chai Y.-F., Yao Y.-M. (2014). Macrophage Polarization in Inflammatory Diseases. Int. J. Biol. Sci..

[7] Badylak S.F., Valentin J.E., Ravindra A.K., McCabe G.P., Stewart-Akers A.M. (2008). Macrophage Phenotype as a Determinant of Biologic Scaffold Remodeling. Tissue Eng. Part A.

[8] Yahfoufi N., Alsadi N., Jambi M., Matar C. (2018). The Immunomodulatory and Anti-Inflammatory Role of Polyphenols. Nutrients.

[9] Kumar S., Pandey A.K. (2013). Chemistry and Biological Activities of Flavonoids: An Overview. Sci. World J..

[10] Maleki S.J., Crespo J.F., Cabanillas B. (2019). Anti-inflammatory effects of flavonoids. Food Chem..

[11] Saqib U., Sarkar S., Suk K., Mohammad O., Baig M.S., Savai R. (2018). Phytochemicals as modulators of M1-M2 macrophages in inflammation. Oncotarget.

[12] Hosseinzade A., Sadeghi O., Biregani A.N., Soukhtehzari S., Brandt G.S., Esmaillzadeh A. (2019). Immunomodulatory Effects of Flavonoids: Possible Induction of T CD4+ Regulatory Cells Through Suppression of mTOR Pathway Signaling Activity. Front. Immunol..

[13] Mendes L.F., Gaspar V.M., Conde T.A., Mano J.F., Duarte I.F. (2019). Flavonoid-mediated immunomodulation of human macrophages involves key metabolites and metabolic pathways. Sci. Rep..

[14] Tyurina Y.Y., Croix C.M.S., Watkins S.C., Watson A.M., Epperly M.W., Anthonymuthu T.S., Kisin E.R., Vlasova I.I., Krysko O., Krysko D.V. (2019). Redox (phospho)lipidomics of signaling in inflammation and programmed cell death. J. Leukoc. Biol..

[15] Katafuchi T., Ifuku M., Mawatari S., Noda M., Miake K., Sugiyama M., Fujino T. (2012). Effects of plasmalogens on systemic lipopolysaccharide-induced glial activation and β-amyloid accumulation in adult mice. Ann. N. Y. Acad. Sci..

[16] Broniec A., Żądło A.C., Pawlak A., Fuchs B., Kłosiński R., Thompson D., Sarna T. (2017). Interaction of plasmenylcholine with free radicals in selected model systems. Free. Radic. Biol. Med..

[17] Vergadi E., Ieronymaki E., Lyroni K., Vaporidi K., Tsatsanis C. (2017). Akt Signaling Pathway in Macrophage Activation and M1/M2 Polarization. J. Immunol..

[18] Rouzer C.A., Ivanova P.T., Byrne M.O., Milne S.B., Marnett L.J., Brown H.A. (2006). Lipid Profiling Reveals Arachidonate Deficiency in RAW264 7 Cells: Structural and Functional Implications. Biochemistry.

[19] Zhang C., Wang Y., Wang F., Wang Z., Lu Y., Xu Y., Wang K., Shen H., Yang P., Li S. (2017). Quantitative profiling of glycerophospholipids during mouse and human macrophage differentiation using targeted mass spectrometry. Sci. Rep..

[20] Lee J.W., Mok H.J., Lee D.Y., Park S.C., Kim G.-S., Lee S.-E., Lee Y.-S., Kim K.P., Kim H.D. (2017). UPLC-QqQ/MS-Based Lipidomics Approach To Characterize Lipid Alterations in Inflammatory Macrophages. J. Proteome Res..

[21] Montenegro-Burke J.R., Sutton J.A., Rogers L.M., Milne G.L., McLean J.A., Aronoff D.M. (2016). Lipid profiling of polarized human monocyte-derived macrophages. Prostaglandins Other Lipid Mediat..

[22] Kanno K., Wu M.K., Scapa E.F., Roderick S.L., David E. (2009). Structure and Function of Phosphatidylcholine Transfer Protein (PC-TP)/StarD2. Structure.

[23] Furse S., De Kroon A.I.P.M. (2015). Phosphatidylcholine’s functions beyond that of a membrane brick. Mol. Membr. Biol..

[24] Penas F., Mirkin G.A., Vera M., Cevey Á., Gonzalez C.D., Gómez M.I., Sales M.E., Goren N.B. (2015). Treatment in vitro with PPARα and PPARγ ligands drives M1-to-M2 polarization of macrophages from T. cruzi-infected mice. Biochim. Biophys. Acta Mol. Basis Dis..

[25] Erridge C., Kennedy S., Spickett C.M., Webb D.J. (2008). Oxidized Phospholipid Inhibition of Toll-like Receptor (TLR) Signaling Is Restricted to TLR2 and TLR4. J. Biol. Chem..

[26] Freigang S. (2016). The regulation of inflammation by oxidized phospholipids. Eur. J. Immunol..

[27] Jiménez-Rojo N., Riezman H. (2019). On the road to unraveling the molecular functions of ether lipids. FEBS Lett..

[28] Watanabe N., Suzuki T., Yamazaki Y., Sugiyama K., Koike S., Nishimukai M. (2019). Supplemental feeding of phospholipid-enriched alkyl phospholipidfrom krill relieves spontaneous atopic dermatitis andstrengthens skin intercellular lipid barriers in NC/Ngamice. Biosci. Biotechnol. Biochem..

[29] Dean J.M., Lodhi I.J. (2017). Structural and functional roles of ether lipids. Protein Cell.

[30] Qin X., Qiu C., Zhao L. (2014). Lysophosphatidylcholine perpetuates macrophage polarization toward classically activated phenotype in inflammation. Cell. Immunol..

[31] Ross T., Jakubzig B., Grundmann M., Massing U., Kostenis E., Schlesinger M., Bendas G. (2016). The molecular mechanism by which saturated lysophosphatidylcholine attenuates the metastatic capacity of melanoma cells. FEBS Open Biol..

[32] Andrejeva G., Rathmell J.C. (2017). Similarities and Distinctions of Cancer and Immune Metabolism in Inflammation and Tumors. Cell Metab..

[33] O’Donnell V.B., Rossjohn J., Wakelam M.J.O. (2018). Phospholipid signaling in innate immune cells. J. Clin. Investig..

[34] Gardocki M.E., Jani N., Lopes J.M. (2005). Phosphatidylinositol biosynthesis: Biochemistry and regulation. Biochim. Biophys. Acta Mol. Basis Dis..

[35] Vivanco I., Sawyers C.L. (2002). The phosphatidylinositol 3-kinase-AKT pathway in humancancer. Nat. Rev. Cancer.

[36] Stark A.-K., Sriskantharajah S., Hessel E.M., Okkenhaug K. (2015). PI3K inhibitors in inflammation, autoimmunity and cancer. Curr. Opin. Pharmacol..

[37] Yeom M., Hahm D.-H., Sur B.-J., Han J.-J., Lee H., Yang H.-I., Kim K.S. (2013). Phosphatidylserine inhibits inflammatory responses in interleukin-1β–stimulated fibroblast-like synoviocytes and alleviates carrageenan-induced arthritis in rat. Nutr. Res..

[38] Maceyka M., Spiegel S. (2014). Sphingolipid metabolites in inflammatory disease. Nature.

[39] Lou B., Dong J., Li Y., Ding T., Bi T., Li Y., Deng X., Ye D., Jiang X.-C. (2014). Pharmacologic Inhibition of Sphingomyelin Synthase (SMS) Activity Reduces Apolipoprotein-B Secretion from Hepatocytes and Attenuates Endotoxin-Mediated Macrophage Inflammation. PLoS ONE.

[40] Norris G.H., Blesso C.N. (2017). Dietary and Endogenous Sphingolipid Metabolism in Chronic Inflammation. Nutrients.

[41] Viola A., Munari F., Sánchez-Rodríguez R., Scolaro T., Castegna A. (2019). The Metabolic Signature of Macrophage Responses. Front. Immunol..

[42] Kanaze F.I., Bounartzi M.I., Georgarakis M., Niopas I. (2006). Pharmacokinetics of the citrus flavanone aglycones hesperetin and naringenin after single oral administration in human subjects. Eur. J. Clin. Nutr..

[43] Dabeek W., Marra M.V. (2019). Dietary Quercetin and Kaempferol: Bioavailability and Potential Cardiovascular-Related Bioactivity in Humans. Nutrients.

[44] Dyer W.J., Bligh E.G. (1963). A rapid method of total lipid extraction and purification. Can. J. Biochem. Physiol..

[45] Bartlett E.M., Lewis D.H. (1970). Spectrophotometric determination of phosphate esters in the presence and absence of orthophosphate. Anal Biochem..

[46] Colombo S., Melo T., Martínez-López M., Carrasco M.J., Domingues M.D.R., Pérez-Sala D., Domingues P. (2018). Phospholipidome of endothelial cells shows a different adaptation response upon oxidative, glycative and lipoxidative stress. Sci. Rep..

[47] Anjos S., Feiteira E., Cerveira F., Melo T., Reboredo A., Colombo S., Dantas R., Costa E., Moreira A., Santos S. (2019). Lipidomics Reveals Similar Changes in Serum Phospholipid Signatures of Overweight and Obese Pediatric Subjects. J. Proteome Res..

[48] Pluskal T., Castillo S., Villar-Briones A., Orešič M. (2010). MZmine 2: Modular framework for processing, visualizing, and analyzing mass spectrometry-based molecular profile data. BMC Bioinform..

[49] Xia J., Sinelnikov I.V., Han B., Wishart D.S. (2015). MetaboAnalyst 3.0—making metabolomics more meaningful. Nucleic Acids Res..

