# Supplementary material for: Differential Modulation of the Phospholipidome of Proinflammatory Human Macrophages by the Flavonoids Quercetin, Naringin and Naringenin
Source: Molecules. 2020 Jul 29;25(15):3460. doi: 10.3390/molecules25153460 (PMC7436172; doi:10.3390/molecules25153460)
Supplement: Supplementary file 1 [file molecules-25-03460-s001.pdf]

Supplementary Information

Differential modulation of the phospholipidome of pro-inflammatory human macrophages by the flavonoids quercetin, naringin and naringenin

Tiago A. Conde[1,2], Luís Mendes[1], Vítor M. Gaspar[1], João F. Mano[1], Tânia Melo[2,3], M. Rosário Domingues[2,3,]*, Iola F. Duarte[1,]*

[1]CICECO-Aveiro Institute of Materials, Department of Chemistry, University of Aveiro, 3810-193 Aveiro, Portugal.
[2]LAQV-REQUIMTE, Mass Spectrometry Center, Department of Chemistry, University of Aveiro, 3810-193 Aveiro, Portugal.
[3]CESAM, Department of Chemistry, University of Aveiro, 3810-193 Aveiro, Portugal.

*Corresponding authors: Iola F. Duarte, ioladuarte@ua.pt; M. Rosário Domingues, mrd@ua.pt

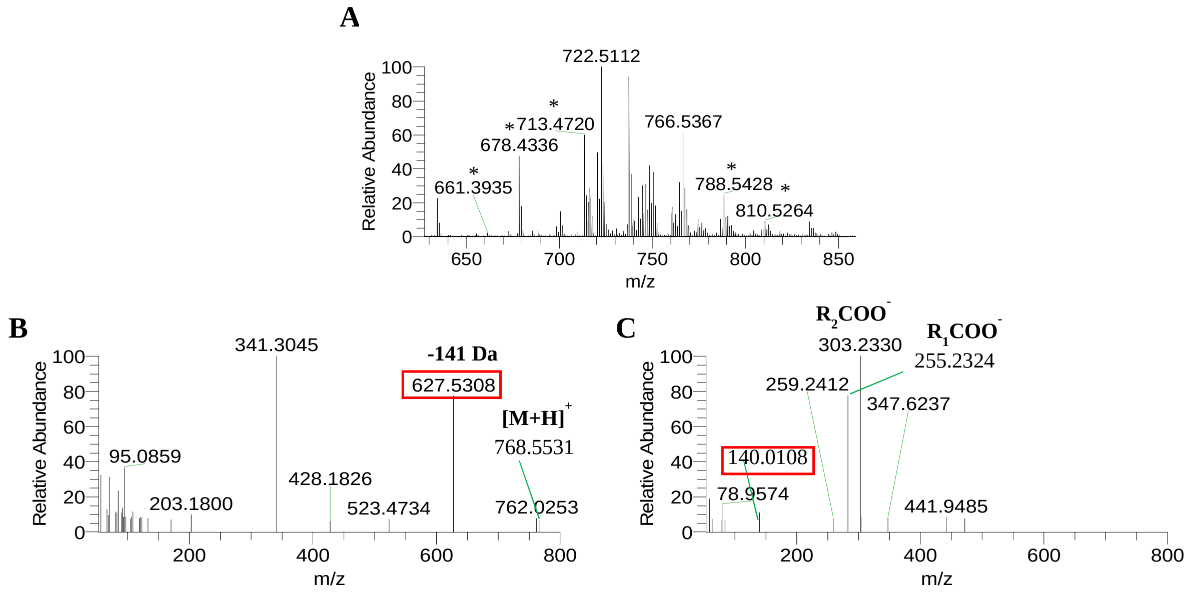

Figure S1. Identification of phosphatidylethanolamine (PE). (A) MS spectrum of [M-H]- ions of PE species. (B) ESI-MS/MS spectrum (HCD fragmentation) of the [M+H]+ ion of PE(38:4) (m/z 768.5531). (C) ESI-MS/MS spectrum (HCD) fragmentation of the [M-H]- ion of PE(38:4) (m/z 766.5367). Fragment ions characteristic for the PE class were highlighted in a red box. *background

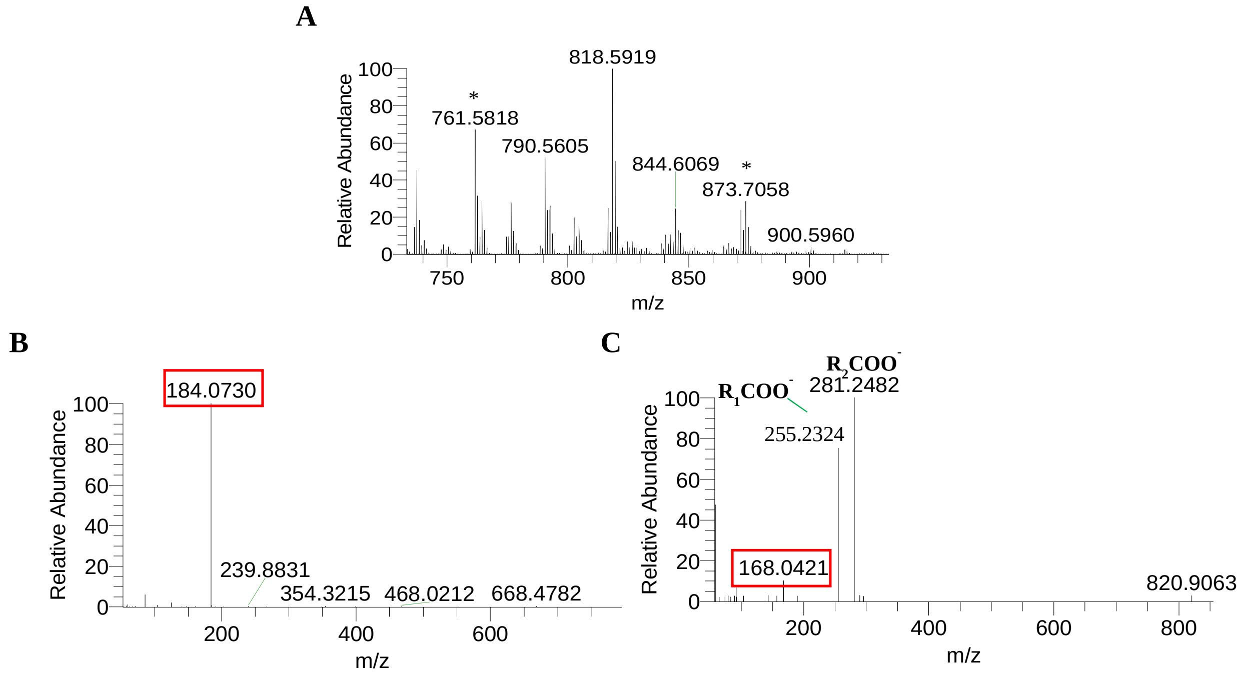

Figure S2. Identification of phosphatidylcholine (PC). (A) MS spectrum of [M+CH3COO]- ions of PC species. (B) ESI-MS/MS spectrum (HCD fragmentation) of the [M+H]+ ion of PC(34:1) (m/z 760.5848). (C) ESI-MS/MS spectrum (HCD) fragmentation of the [M+CH3COO]- ion of PC(34:1) (m/z 818.5908). Fragment ions characteristic for the PC class were highlighted in a red box. *background

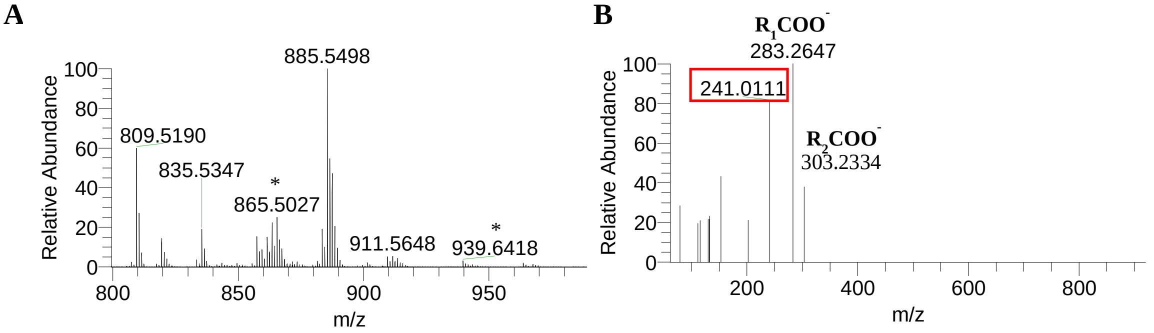

Figure S3. Identification of phosphatidylinositol (PI). (**A**) MS spectrum of [M-H]$^-$ ions of PI species. (**B**) ESI-MS/MS spectrum (HCD) fragmentation of the [M-H]$^-$ ion of PI(38:4) (*m/z* 885.5488). Fragment ions characteristic for the PI class were highlighted in a red box. *background

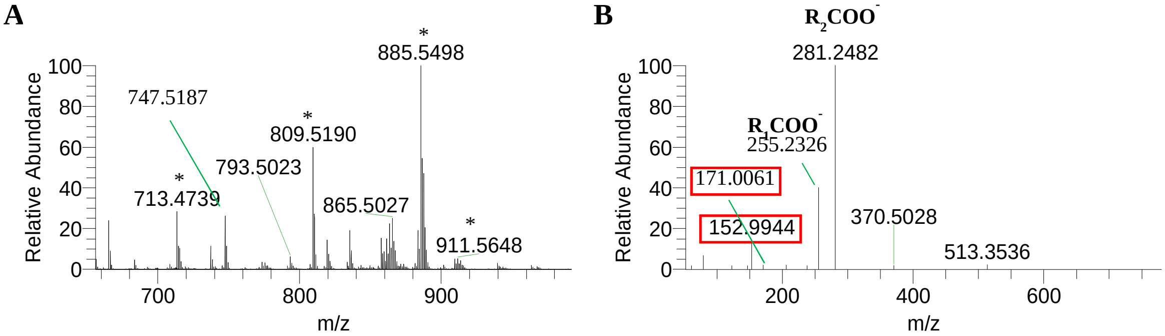

Figure S4. Identification of phosphatidylglycerol (PG). (**A**) MS spectrum of [M-H]$^-$ ions of PG species. (**B**) ESI-MS/MS spectrum (HCD) fragmentation of the [M-H]$^-$ ion of PG(34:1) (*m/z* 747.5174). Fragment ions characteristic for the PG class were highlighted in a red box. *background

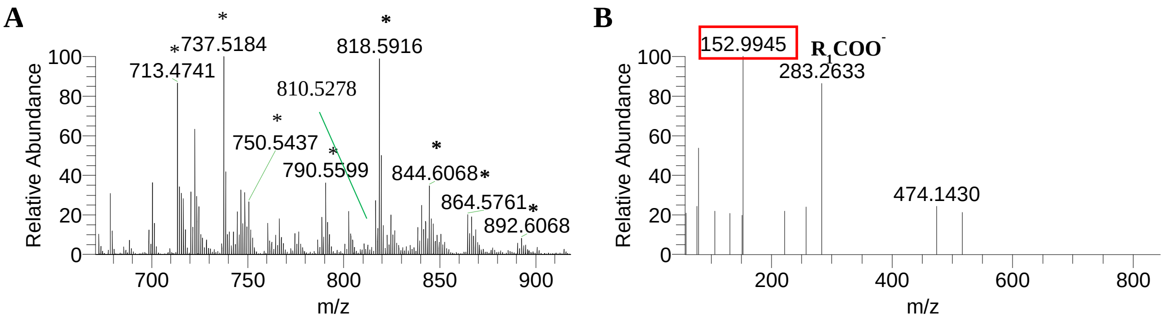

Figure S5. Identification of phosphatidylserine (PS). (**A**) MS spectrum of $[M\text{-}H]^-$ ion of PS species. (**B**) ESI-MS/MS spectrum (HCD fragmentation) of the $[M\text{-}H]^-$ ion of PS(38:4) (*m/z* 810.5278). Fragment ions characteristic for the PS class were highlighted in a red box. *background

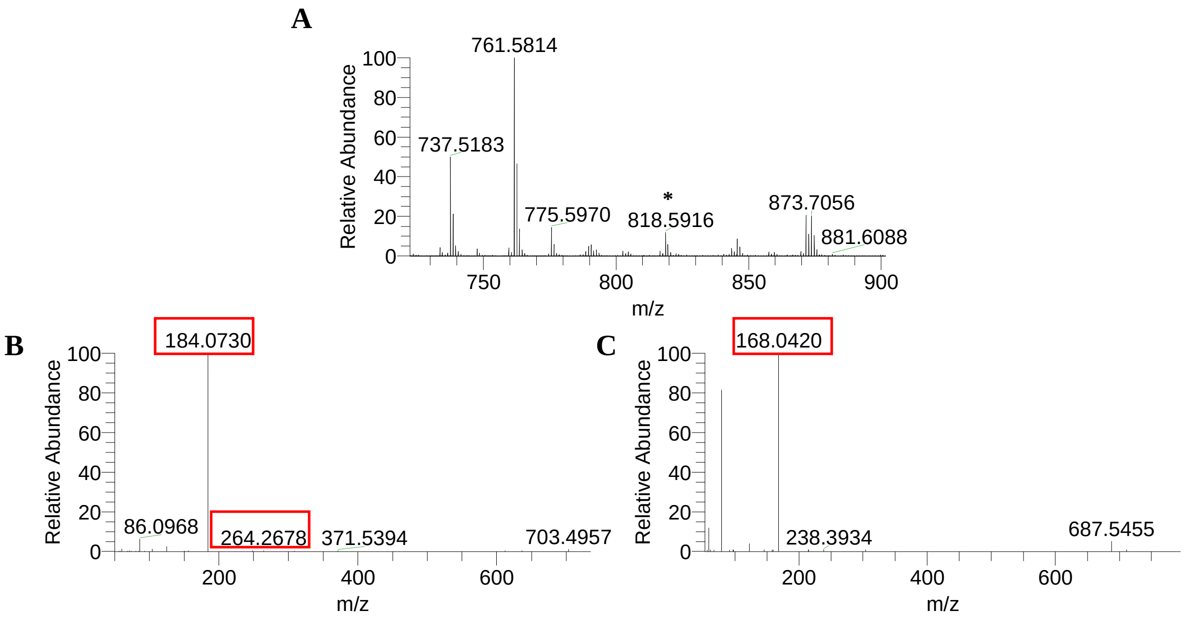

Figure S6. Identification of sphingomyelin (SM). (**A**) MS spectrum of $[M+CH_3COO]^-$ ions of SM species. (**B**) ESI-MS/MS spectrum (HCD fragmentation) of the $[M+H]^+$ ion of SM(d34:1) (m/z 703.5750). (**C**) ESI-MS/MS spectrum (HCD) fragmentation of the $[M+CH_3COO]^-$ ion of SM(d34:1) (m/z 761.5814). Fragment ions characteristic for the SM class were highlighted in a red box. *background

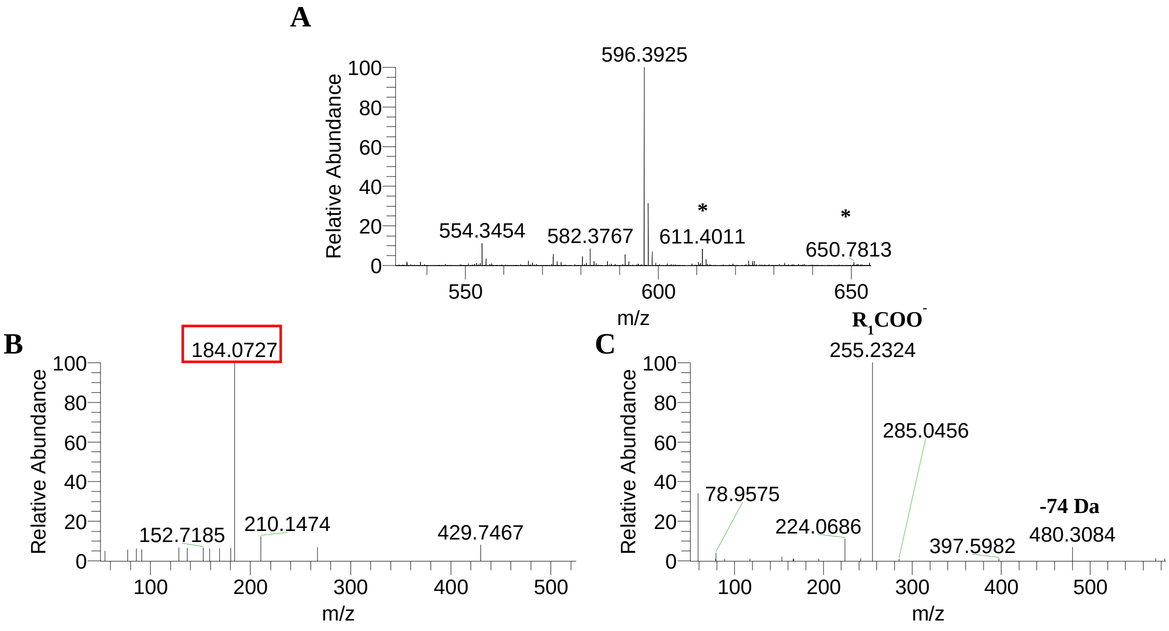

Figure S7. Identification of lyso-phosphatidylcholine (LPC). (**A**) MS spectrum of $[M+CH_3COO]^-$ ions of LPC species. (**B**) ESI-MS/MS spectrum (HCD fragmentation) of the $[M+H]^+$ ion of LPC(16:0) (*m/z* 496.3398). (**C**) ESI-MS/MS spectrum (HCD) fragmentation of the $[M+CH_3COO]^-$ ion of LPC(16:0) (*m/z* 554.3460). Fragment ions characteristic for the LPC class were highlighted in a red box. *background

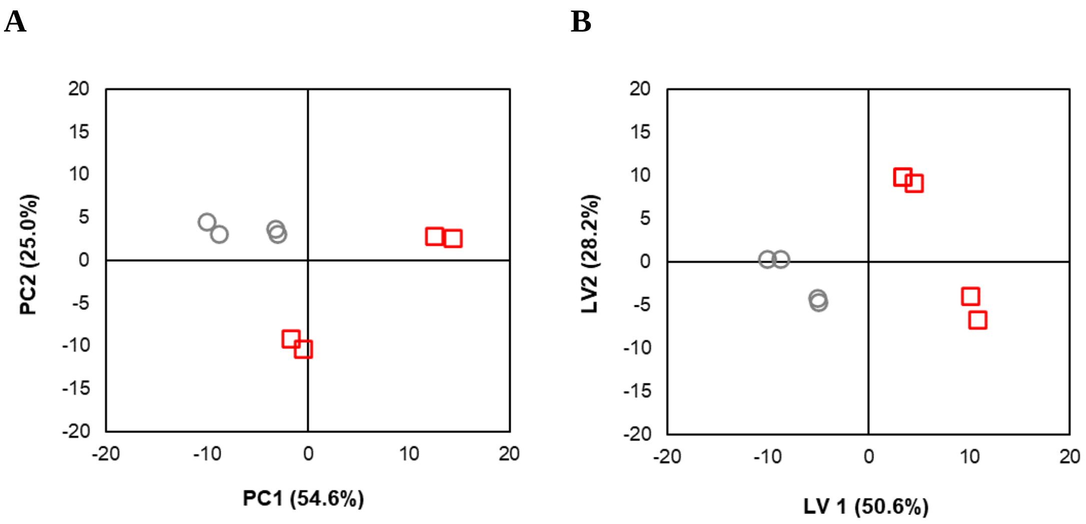

Figure S8. Multivariate analysis of LC-MS data collected for M0 (grey circles) and M1 (red squares) macrophages: (A) PCA and (B) PLS-DA scores scatter plots.

Table S1. Phospholipid molecular species identified in THP-1-derived macrophages.

Lipid species (C:N)	Calculated *m/z*	Observed *m/z*	Error (ppm)	Fatty acyl chains (C:N)	Formula
**LPC identified as [M+CH3COO]$^-$**					
LPC(16:0)	554,3455	554,3458	**0,4755**	**16:0**	C26H53NO9P
LPC(18:0)	582,3770	582,3771	**0,1904**	**18:0**	C28H57NO9P
LPC(18:1)	580,3614	580,3614	**0,0639**	**18:1**	C28H55NO9P
**LPE identified as [M-H]-**					
LPE(16:0)	452,2779	452,2777	**-0,5021**	**16:0**	C21H43NO7P
LPE(18:0)	480,3093	480,3090	**-0,5484**	**18:0**	C23H47NO7P
LPE(18:1)	478,2931	478,2934	**0,6425**	**18:1**	C23H45NO7P
LPE(18:2)	476,2774	476,2777	**0,6402**	**18:2**	C23H43NO7P
LPE(20:3)	502,2939	502,2934	**-1,1113**	**20:3**	C25H45NO7P
LPE(20:4)	500,2778	500,2777	**-0,0800**	**20:4**	C25H43NO7P
LPE(20:5)	498,2619	498,2621	**0,3767**	**20:5**	C25H41NO7P
LPE(22:5)	526,2933	526,2934	**0,0517**	**22:5**	C27H45NO7P
LPE(22:6)	524,2774	524,2777	**0,5377**	**22:6**	C27H43NO7P
**PC identified as [M+CH3COO]$^-$**					
PC(30:0)	764,5442	764,5447	**0,6631**	**14:0/16:0**	C40H79NO10P
PC(30:1)	762,5285	762,5287	**0,2465**	**14:0/16:1 and 14:1/16:0**	C40H77NO10P
PC(32:0)	792,5755	792,5753	**-0,2333**	**16:0/16:0**	C42H83NO10P
PC(32:1)	790,5598	790,5584	**-1,7588**	**14:0/18:1 and 16:0/16:1**	C42H81NO10P
PC(32:2)	788,5442	788,5434	**-0,9330**	**16:0/16:2 and 14:0/18:2**	C42H79NO10P
PC(34:1)	818,5911	818,5888	**-2,8451**	**16:0/18:1**	C44H85NO10P
PC(34:2)	816,5755	816,5755	**0,0696**	**16:0/18:2 and 16:1/18:1**	C44H83NO10P
PC(34:3)	814,5598	814,5601	**0,3822**	**16:0/18:3 and 16:1/18:2**	C44H81NO10P
PC(34:4)	812,5442	812,5418	**-2,9035**	**	C44H79NO10P
PC(36:1)	846,6224	846,6196	**-3,2861**	**18:0/18:1**	C46H89NO10P
PC(36:2)	844,6068	844,6063	**-0,4993**	**18:1/18:1**	C46H87NO10P
PC(36:3)	842,5911	842,5909	**-0,2680**	**16:0/20:3 and 18:1/18:2**	C46H85NO10P
PC(36:4)	840,5755	840,5728	**-3,1724**	**16:0/20:4 and 16:1/20:3**	C46H83NO10P
PC(36:5)	838,5598	838,5590	**-0,9799**	**16:0/20:5 and 16:1/20:4**	C46H81NO10P
PC(36:6)	836,5442	836,5422	**-2,2891**	**16:1/20:5**	C46H79NO10P
PC(38:3)	870,6224	870,6213	**-1,2845**	**16:0/22:3, 18:0/20:3, 18:1/20:2 and 18:2/20:1**	C48H89NO10P
PC(38:4)	868,6068	868,6041	**-3,0474**	**18:0/20:4**	C48H87NO10P
PC(38:5)	866,5911	866,5913	**0,1832**	**18:1/20:4**	C48H85NO10P
PC(38:6)	864,5755	864,5745	**-1,0878**	**16:0/22:6**	C48H83NO10P
PC(38:7)	862,5598	862,5587	**-1,3147**	**18:0/22:7**	C48H81NO10P
PC(40:4)	896,6381	896,6349	**-3,4845**	**18:1/22:3 and 18:2/22:2**	C50H91NO10P
PC(40:5)	894,6224	894,6224	**-0,0355**	**	C50H89NO10P
PC(40:6)	892,6068	892,6043	**-2,7152**	**18:1/22:5**	C50H87NO10P

Supplementary Table 1 (Cont.)

Lipid species (C:N)	Calculated *m/z*	Observed *m/z*	Error (ppm)	Fatty acyl chains (C:N)	Formula
PC(40:7)	890,5911	890,5897	**-1,5679**	**18:1/22:6**	C50H85NO10P
PC(O-28:0)	722,5336	722,5335	**-0,1785**	**	C38H77NO9P
PC(O-30:0)	750,5649	750,5634	**-1,9413**	**14:0/16:0 and 16:0/14:0**	C40H81NO9P
PC(O-30:1)/PC(P-30:0)	748,5492	748,5483	**-1,2073**	**14:0/16:1 and 16:0/14:1**	C40H79NO9P
PC(O-32:0)	778,5962	778,5947	**-1,9795**	**	C42H85NO9P
PC(O-32:1)/PC(P-32:0)	776,5805	776,5792	**-1,7513**	**16:0/16:1 and 16:1/16:0**	C42H83NO9P
PC(O-34:1)/PC(P-34:0)	804,6118	804,6093	**-3,1557**	**16:0/18:1 and 18:1/16:0**	C44H87NO9P
PC(O-34:2)/PC(P-34:1)	802,5962	802,5944	**-2,2724**	**16:0/18:2, 16:1/18:1, 18:1/16:1 and 18:2/16:0**	C44H85NO9P
PC(O-34:3)/PC(P-34:2)	800,5805	800,5808	**0,3135**	**18:2/16:1**	C44H83NO9P
PC(O-36:1)/ PC(P-36:0)	832,6431	832,6432	**0,0440**	**18:1/18:0**	C46H91NO9P
PC(O-36:2)/PC(P-36:1)	830,6275	830,6252	**-2,7152**	**18:1/18:1**	C46H89NO9P
PC(O-36:3)/PC(P-36:2)	828,6118	828,6107	**-1,3701**	**18:1/18:2, 18:2/18:1 and 20:3/16:0**	C46H87NO9P
PC(O-36:5)/PC(P-36:4)	824,5805	824,5796	**-1,1001**	**20:4/16:1**	C46H83NO9P
PC(O-38:4)/ PC(P-38:3)	854,6275	854,6247	**-3,2888**	**20:4/18:0 and 20:3/18:1**	C48H89NO9P
PC(O-38:5)/PC(P-38:4)	852,6118	852,6102	**-1,9716**	**20:4/18:1**	C48H87NO9P
PC(O-38:6)/PC(P-38:5)	850,5962	850,5943	**-2,1765**	**16:1/22:5, 18:0/20:6 and 22:6/16:0**	C48H85NO9P
PC(O-40:5)/PC(P-40:4)	880,6431	880,6408	**-2,6434**	**20:4/20:1**	C50H91NO9P
PC(O-42:4)/PC(P-42:3)	910,6901	910,6882	**-2,0772**	**20:4/22:0**	C52H97NO9P
PC(O-42:5)/PC(P-42:4)	908,6744	908,6745	**0,0779**	**	C52H95NO9P
PC(P-32:1)	774,5649	774,5634	**-1,9079**	**16:1/16:1**	C42H81NO9P
PC(P-36:5)	822,5649	822,5667	**2,1702**	**20:5/16:1**	C46H81NO9P
PC(P-38:6)	848,5805	848,5795	**-1,1941**	**18:1/20:5 and 18:0/20:6**	C48H83NO9P
PC(P-40:6)	876,6118	876,6099	**-2,2685**	**	C50H87NO9P
			**PE identified as [M-H]$^{-}$**		
PE(32:0)	690,5050	690,5074	**3,4798**	*	C37H73NO8P
PE(32:1)	688,4911	688,4917	**0,9429**	**16:0/16:1**	C37H71NO8P
PE(34:1)	716,5223	716,5230	**1,0321**	**16:1/18:0 and 16:0/18:1**	C39H75NO8P
PE(34:2)	714,5077	714,5074	**-0,4095**	**16:1/18:1 and 16:0/18:2**	C39H73NO8P
PE(36:1)	744,5542	744,5543	**0,1327**	**18:0/18:1**	C41H79O8NP
PE(36:2)	742,5372	742,5387	**1,9930**	**18:1/18:1 and 18:0/18:2**	C41H77O8NP
PE(36:5)	736,4904	736,4917	**1,8313**	**16:0/20:5 and 16:1/20:4**	C41H71O8NP
PE(38:3)	768,5524	768,5543	**2,5380**	**18:0/20:3**	C43H79NO8P
PE(38:4)	766,5379	766,5367	**-1,5655**	**18:0/20:4**	C43H77O8NP
PE(38:5)	764,5221	764,5230	**1,2435**	**18:1/20:4 and 18:0/20:5**	C43H75NO8P
PE(38:6)	762,5087	762,5074	**-1,6821**	**16:0/22:6 and 18:1/20:5**	C43H73O8NP
PE(40:4)	794,5687	794,5700	**1,6131**	**18:2/22:2**	C45H81NO8P
PE(40:5)	792,5531	792,5543	**1,5114**	**	C45H79O8NP
PE(40:6)	790,5375	790,5387	**1,4475**	**18:0/22:6**	C45H77O8NP
PE(O-32:2)/PE(P-32:1)	672,4966	672,4968	**0,3567**	**16:1/16:1**	C37H71O7NP

Supplementary Table 1 (Cont.)

Lipid species (C:N)	Calculated *m/z*	Observed *m/z*	Error (ppm)	Fatty acyl chains (C:N)	Formula
PE(O-32:3)/PE(P-32:2)	670,4818	670,4812	**-0,9665**	**	C37H69O7NP
PE(O-34:2)/PE(P-34:1)	700,5281	700,5281	**0,0531**	**16:1/18:1, 16:0/18:2, 18:1/16:1 and 18:2/16:0**	C39H75NO7P
PE(O-34:3)/PE(P-34:2)	698,5116	698,5125	**1,2949**	**18:2/16:1**	C39H73NO7P
PE(O-34:4)/PE(P-34:3)	696,4978	696,4968	**-1,4398**	**18:3/16:1 and 18:1/16:2**	C39H71NO7P
PE(O-34:5)/PEp(34:4)	694,4799	694,4812	**1,8002**	**20:4/14:1**	C39H69O7NP
PE(O-36:2)/PE(P-36:1)	728,5582	728,5594	**1,7134**	**18:1/18:1**	C41H79NO7P
PE(O-36:3)PE(P-36:2)	726,5433	726,5438	**0,6971**	**18:1/18:2 and 18:2/18:1**	C41H77NO7P
PE(O-36:4)/PE(P-36:3)	724,5275	724,5281	**0,8448**	**	C41H75NO7P
PE(O-36:5)/PE(P-36:4)	722,5118	722,5125	**0,8599**	**20:4/16:0**	C41H73NO7P
PE(O-36:6)/PE(P-36:5)	720,4963	720,4968	**0,7853**	**20:5/16:1**	C41H71O7NP
PE(O-38:5)/PE(P-38:4)	750,5425	750,5438	**1,6653**	**20:3/18:2, 20:4/18:1, 20:5/18:0 and 22:4/16:1**	C43H77NO7P
PE(O-38:6)/PE(P-38:5)	748,5264	748,5281	**2,3491**	**20:4/18:2, 20:5/18:1 and 22:4/16:1**	C43H75NO7P
PE(P-38:6)	746,5121	746,5125	**0,4529**	**22:6/16:1**	C43H73O7NP
PE(O-40:4)/PE(P-40:3)	780,5899	780,5907	**1,0527**	**	C45H83NO7P
PE(O-40:5)/PE(P-40:4)	778,5731	778,5751	**2,5602**	**	C45H81NO7P
PE(O-40:6)/PE(P-40:5)	776,5599	776,5594	**-0,5948**	**	C45H79NO7P
PE(P-40:6)	774,5422	774,5438	**2,0109**	**	C45H77O7NP
PE(P-40:7)	772,5262	772,5281	**2,5021**	**22:6/18:2**	C45H75O7NP
PE(O-42:5)/PE(P-42:4)	806,6038	806,6064	**3,1809**	**20:3/22:2**	C47H85O7NP
PE(O-42:6)/PE(P-42:5)	804,5902	804,5907	**0,6666**	**20:4/22:2**	C47H83O7NP
PE(P-42:6)	802,5742	802,5751	**1,0630**	**20:5/22:4**	C47H81O7NP
**PG identified as [M-H]$^-$**					
PG(32:1)	719,4850	719,4863	**1,8758**	**16:0/16:1 and 14:0/18:1**	C38H72O10P
PG(34:1)	747,5172	747,5176	**0,5169**	**16:0/18:1**	C40H76O10P
PG(34:2)	745,5005	745,5020	**1,8998**	**16:1/18:1 and 16:0/18:2**	C40H74O10P
PG(36:1)	775,5486	775,5489	**0,3628**	*	C42H80O10P
PG(36:2)	773,5309	773,5333	**3,0111**	**18:1/18:1**	C42H78O10P
PG(36:3)	771,5165	771,5176	**1,3925**	**	C42H76O10P
PG(36:4)	769,4994	769,5020	**3,2777**	**	C42H74O10P
PG(38:4)	797,5318	797,5333	**1,8597**	**18:1/20:3**	C44H78O10P
PG(38:5)	795,5172	795,5176	**0,5386**	*	C44H76O10P
PG(38:6)	793,5003	793,5020	**2,1095**	**16:0/22:6 and 18:0/20:6**	C44H74O10P
PG(38:7)	791,4861	791,4863	**0,2925**	**16:1/22:6**	C44H72O10P
PG(40:7)	819,5161	819,5176	**1,8256**	**18:1/22:6**	C46H76O10P
PG(42:10)	841,5016	841,5020	**0,4182**	**20:4/22:6**	C48H74O10P
PG(44:12)	865,5006	865,5020	**1,5970**	**22:6/22:6**	C50H74O10P
**PI identified as [M-H]$^-$**					
PI(32:1)	807,5019	807,5024	**0,5280**	**16:0/16:1**	C41H76O13P
PI(34:1)	835,5334	835,5337	**0,3223**	**16:0/18:1 and 16:1/18:0**	C43H80O13P
PI(34:2)	833,5161	833,5180	**2,2643**	**16:0/18:2 and 16:1/18:1**	C43H78O13P
PI(36:1)	863,5630	863,5650	**2,2416**	**18:0/18:1**	C45H84O13P
PI(36:2)	861,5489	861,5493	**0,5287**	**18:1/18:1, 18:0/18:2 and 16:0/20:2**	C45H82O13P

PI(36:3)	859,5336	859,5337	**0,0875**	**	C45H80O13P
PI(36:4)	857,5174	857,5180	**0,6807**	**16:0/20:4**	C45H78O13P
PI(36:5)	855,5005	855,5024	**2,1891**	**16:0/20:5**	C45H76O13P
PI(38:4)	885,5487	885,5493	**0,6412**	**18:0/20:4**	C47H82O13P
PI(38:5)	883,5309	883,5337	**3,1067**	**18:1/20:4 and 18:0/20:5**	C47H80O13P
PI(38:6)	881,5163	881,5180	**1,9126**	**16:0/22:6**	C47H78O13P
PI(40:3)	915,5946	915,5963	**1,8068**	**	C49H88O13P
PI(40:4)	913,5792	913,5806	**1,5046**	**18:0/22:4**	C49H86O13P
PI(40:5)	911,5656	911,5650	**-0,6823**	**18:0/22:5**	C49H84O13P
PI(40:6)	909,5473	909,5493	**2,2401**	**18:0/22:6 and 18:1/22:5**	C49H82O13P
PI(40:7)	907,5320	907,5337	**1,8724**	**18:1/22:6**	C49H80O13P
		**PS identified as $[M-H]^-$**			
PS(32:1)	732,4812	732,4816	**0,5496**	**16:0/16:1**	C38H71NO10P
PS(34:1)	760,5112	760,5129	**2,2047**	**16:0/18:1 and 16:1/18:0**	C40H75NO10P
PS(34:2)	758,4955	758,4972	**2,2935**	**16:0/18:2**	C40H73NO10P
PS(36:1)	788,5433	788,5442	**1,1095**	**18:0/18:1**	C42H79NO10P
PS(36:2)	786,5263	786,5285	**2,8574**	**18:0/18:2**	C42H77NO10P
PS(36:3)	784,5115	784,5129	**1,6923**	**	C42H75NO10P
PS(36:4)	782,4969	782,4972	**0,4135**	**	C42H73NO10P
PS(38:3)	812,5417	812,5442	**3,0853**	**18:0/20:3**	C44H79NO10P
PS(38:4)	810,5289	810,5285	**-0,5074**	**18:0/20:4**	C44H77NO10P
PS(38:5)	808,5129	808,5129	**-0,0777**	**18:2/20:3**	C44H75NO10P
PS(40:6)	834,5270	834,5285	**1,8138**	**18:0/22:6**	C46H77NO10P
		**SM identified as $[M+CH3COO]^-$**			
SM(d32:1)	733,5496	733,5492	**-0,5039**	*	C39H78N2O8P
SM(d34:1)	761,5809	761,5799	**-1,2386**	**d18:1/16:0**	C41H82N2O8P
SM(d34:2)	759,5652	759,5645	**-0,9840**	*	C41H80N2O8P
SM(d36:1)	789,6122	789,6107	**-1,8237**	*	C43H86N2O8P
SM(d36:2)	787,5965	787,5957	**-1,0015**	*	C43H84N2O8P
SM(d38:1)	817,6435	817,6414	**-2,5599**	*	C45H90N2O8P
SM(d40:1)	845,6748	845,6738	**-1,1689**	*	C47H94N2O8P
SM(d40:2)	843,6591	843,6599	**0,8738**	*	C47H92N2O8P
SM(d42:1)	873,7061	873,7058	**-0,3445**	*	C49H98N2O8P
SM(d42:2)	871,6904	871,6884	**-2,3429**	**d18:1/24:1**	C49H96N2O8P
SM(d42:3)	869,6748	869,6729	**-2,1697**	*	C49H94N2O8P
SM(d44:2)	899,7217	899,7207	**-1,1302**	*	C51H100N2O8P

C – carbons; N – number of double bonds; *identified based on exact mass measurements; ** no FA acyl-chain fragments observed.
